# Nonsyndromic Familial Oligodontia with Multiple Dens Invaginatus: A Case Report of an Unusual Case

**DOI:** 10.1155/2013/983580

**Published:** 2013-11-05

**Authors:** D. P. Vinuth, Poonam Agarwal, Gunjan Dube, S. Abhilash, Pallavi Dube

**Affiliations:** ^1^Department of Oral Pathology and Microbiology, Hitkarini Dental College and Hospital, Jabalpur 482005, India; ^2^Department of Oral Medicine Diagnosis and Radiology, Hitkarini Dental College and Hospital, Jabalpur 482005, India; ^3^Department of Oral and Maxillofacial Surgery, Hitkarini Dental College and Hospital, Jabalpur 482005, India; ^4^Department of Conservative Dentistry, Hitkarini Dental College and Hospital, Jabalpur, Madhya Pradesh 482005, India; ^5^Dube Surgical & Dental Hospital, Jabalpur, Madhya Pradesh 482005, India

## Abstract

Oligodontia is a rare dental anomaly with a prevalence of 0.3% in permanent teeth and much less frequency in the primary dentition. Familial oligodontia represents an absence of varying numbers of primary and/or secondary teeth as an isolated trait. It is a complex and multifactorial condition. Many explanations—evolutionary, genetic, and environmental—have been proposed as the etiology. Simultaneous with oligodontia are often the different positional changes of the existing teeth, their morphology, size, and growth disturbances of the maxillofacial skeleton. Early recognition is vital to provide adequate treatment and prevent squeal. Multidisciplinary referral or consultation is thus important in treatment planning to improve function and esthetics. The present paper reports a rare case of familial oligodontia associated with multiple dense invaginatus and microdontia.

## 1. Introduction

Tooth agenesis is the most common craniofacial malformations. Its prevalence in permanent dentition reaches 20%, and its expressivity ranges from only one tooth, usually a third molar, to the whole dentition [1]. The term “severe hypodontia” (or oligodontia) is defined as agenesis of six or more permanent teeth, excluding the third molar (Schalk van der Weide, 1992) [2]. It is found in about one in 15 hypodontia patients [3]. Oligodontia can be isolated (oligodontia/I), as the only phenotypic alteration in a person, or associated to other alterations as part of a syndrome (oligodontia/S). Isolated, nonsyndromic agenesis can be sporadic or familial, and it may be inherited in Mendelian dominant or recessive autosomal mode or X-linked. Penetrance has been traditionally considered as incomplete but high. The expressivity of the various forms is quite variable, with a wide range of missing teeth [4].

Several factors have been proposed for the etiology of oligodontia [5]. It is basically caused by the complex interactions between genetic, epigenetic, and environmental factors during the long process of dental development [6]. The prevalence of hypodontia has been shown to be higher within the extended family circle than in the general population, which supports a hereditary tendency [4]. Familial oligodontia represents an absence of varying numbers of primary and/or secondary teeth as an isolated trait. A large number of candidate genes have been provided from studies in the mouse, but only a few of them have been identified in human family pedigrees affected by hypodontia. This suggests that in many cases, familial human hypodontia may represent a more complex and multifactorial condition [7]. Oligodontia has serious implications for the patient in terms of masticatory function, malocclusion, speech impairment, and psychological impact. As such, severe hypodontia can have a dramatic effect on a patient's (oral health-related) quality of life [2].

The present report describes an unusual case of nonsyndromic familial oligodontia coexistent with multiple dens invaginatus seen clinically and radiographically. Of interest to the author was the consistent presence of multiple dens invaginatus in the siblings of a family associated with oligodontia. To the authors' best of knowledge, no such case has been reported, and this rarity prompted to report the case.

## 2. Case Report

A 21-year-old male patient was referred to our outpatient department with a chief complaint of several missing teeth. Dental history revealed no evidence of extensive extractions; however, past dental records were not available for verification. The patient's medical history revealed that he was born of a full-term pregnancy and was delivered by forceps. The patient's mother gave no history of exposure to radiation and did not take any medications during pregnancy. Patient was born to nonconsanguineous parents. No history of orofacial trauma or unusual childhood diseases was reported. Drug history was not significant. The family history was positive for missing teeth in the father and two (24 years old male & 16 years old female) of the five siblings.

Physical examination revealed a moderately built and nourished young man with height and weight within the normal range for his age and sex. In view of the oligodontia of permanent teeth, a detailed examination was done to rule out syndromes associated with oligodontia. His hearing and vision appeared to be normal. The hair of the scalp, eyebrows, and eyelashes was normal in distribution, density, and texture. Skin and nails were of normal appearance. Sweating, lacrimal, and salivary secretions were within normal limits. Both history and general examination findings were essentially similar in the affected siblings. The clinical examination of the other available members of the family revealed that the mother and the other two siblings had a full complement of teeth with no associated dental anomalies, while the father was completely edentulous (history of presence of only few permanent teeth since the beginning). None of them had clinical ectodermal abnormalities.

*Sibling 1: 21 Years/Male*

**Figure 1:**
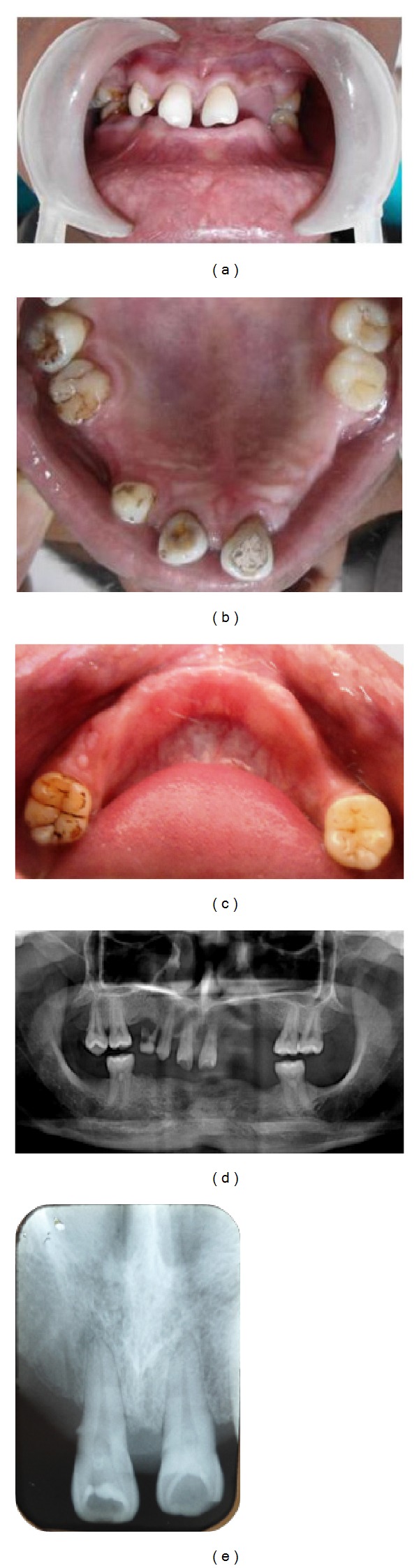
(a) Intraoral photograph of Sibling 1. (b) Intraoral photograph of maxillary arch of Sibling 1. (c) Intraoral photograph of mandibular arch of Sibling 1. (d) OPG of Sibling 1. (e) IOPA of Sibling 1 showing dens invaginatus in both the central incisors.

*Clinical Findings.* Extraoral examination revealed a bilaterally symmetrical face with a straight profile. There was reduction of the lower facial height and protuberance of lips caused by the loss of vertical dimension as a result of oligodontia. Intraorally, soft tissues appeared normal in color and texture. Oral hygiene and gingival status were good and no caries was found. Alveolar bone height was reduced where the teeth were missing. The bone was otherwise healthy and robust looking. Examination of teeth revealed presence of only nine erupted permanent teeth—17, 16, 13, 11, 21, 26, 27, 36, and 46. The remaining permanent teeth were missing clinically (i.e., 19 teeth were congenitally missing excluding the third molars). Of the primary dentition, the retained 54 tooth was present. Abnormal crown morphology was appreciated with 17. The maxillary central incisors were shovel shaped with deep lingual pits. Mild attrition was present with mandibular molars (Figures 1(a), 1(b), and 1(c)).

*Radiographic Findings.* Suspecting the congenital absence of permanent teeth, panoramic radiograph was taken which confirmed congenital absence of all permanent teeth that clinically were not present (Figure 1(d)). Intraoral periapical radiograph was taken in relation to 11 and 21 which revealed the presence of Oehlers type II dens in dente (dens invaginatus) with the central incisors (Figure 1(e)).

*Sibling 2: 16 Years/Female*

**Figure 2:**
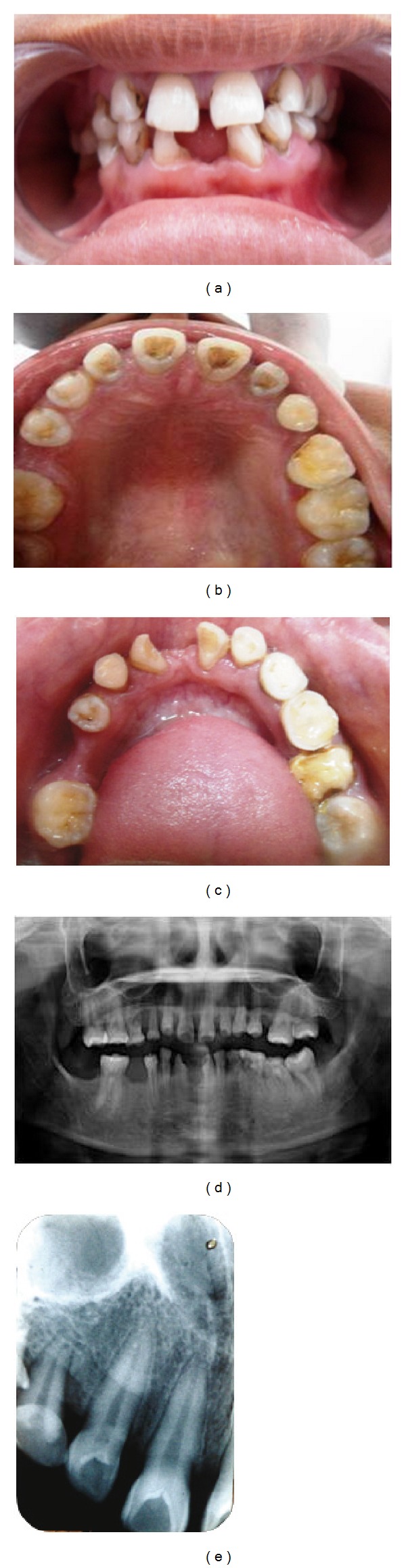
(a) Intraoral photograph of Sibling 2. (b) Intraoral photograph of maxillary arch of Sibling 2. (c) Intraoral photograph of mandibular arch of Sibling 2. (d) OPG of Sibling 2. (e) IOPA of Sibling 2 showing shovel-shaped incisors with dens invaginatus.

*Clinical Findings.* Clinical examination showed facial symmetry with a convex profile. Intraoral examination revealed a faulty prosthesis (fixed with acrylic resin to the adjacent teeth) in the mandibular anterior tooth region. There was moderate localized gingivitis associated with the prosthesis. Hard tissue examination was carried out following the removal of the faulty prosthesis. The bony structure of the upper and lower jaw appeared normal. The following permanent teeth were present and erupted: 17, 16, 12, 11, 21, 22, 24, 25, 26, 27, 36, 37, 32, 42, 44, and 46 (i.e., 12 congenitally missing teeth excluding the third molars). Several retained deciduous teeth were present—55, 53, 73, 74, 75, and 83. The maxillary central and lateral incisors were shovel shaped with deep lingual pits. Mild generalized attrition was present. Mesial rotation was noted with 32 and 42. Generalized microdontia with spacing in the permanent dentition was appreciable (Figures 2(a), 2(b), and 2(c)).

*Radiographic Findings.* Panoramic radiograph disclosed congenital absence of all permanent teeth that were missing on clinical examination with generalized spacing (Figure 2(d)). On periapical radiography, Oehlers type I dens in dente was seen in relation to all maxillary incisors (Figure 2(e)).

*Sibling 3: 24 Years/Male*

**Figure 3:**
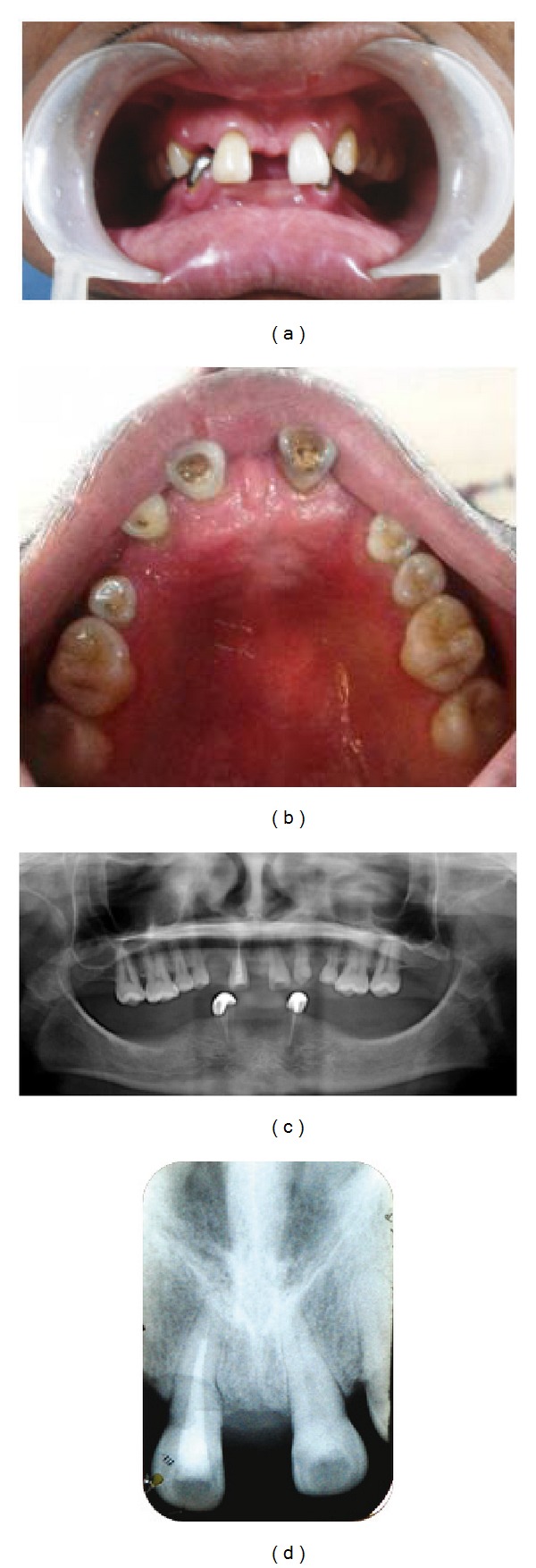
(a) Intraoral photograph of Sibling 3. (b) Intraoral photograph of maxillary arch of Sibling 3. (c) OPG of Sibling 3. (d) IOPA of Sibling 3 showing incisor with dens invaginatus.

*Clinical Findings.* Extraoral examination revealed bilaterally symmetrical face. Patient had a concave profile. There was reduction of the lower facial height and protuberant lips caused by the loss of vertical dimension as a result of oligodontia. Intraoral examination revealed an overdenture with the mandibular arch. Dental history disclosed presence of multiple retained primary teeth in the lower arch which were extracted due to mobility. However, past dental records were not available for verification. Examination of the maxillary arch revealed the presence of following permanent teeth: 17, 16, 15, 14, 11, 21, 24, 25, 26, and 27. Microdontia was present with the premolars. Shovel-shaped crown morphology was appreciable with 21, with associated deep lingual pit (Figures 3(a) and 3(b)).

*Radiographic Findings.* Panoramic radiography confirmed the congenital absence of following permanent teeth: 13, 12, 22, and 23 (Figure 3(c)). Oehlers Type I dens in dente was detected with 21 on periapical radiograph (Figure 3(d)).

Our clinical & radiographic examinations and interviews of the kindred revealed that all affected members were missing more than 6 teeth and had not the other systemic abnormality. They were diagnosed for nonsyndromic oligodontia.

## 3. Discussion

Characteristic dental findings of oligodontia are a reduced number of teeth, smaller teeth, malformation of the teeth, and the delayed eruption [8]. In the present cases, along with oligodontia, the patients exhibited malformation of teeth in the form of dens invaginatus and microdontia. However, no evidence of delayed eruption was noted.

When looking at the association of the number and type of missing teeth amongst siblings, no consistency in pattern was seen. Studies of families suggest that genetic factors may play an important role in oligodontia. Recently investigators favor the hypothesis of a polygenically determined, quasicontinuous type of inheritance. However, environmental factors cannot be ignored. Some of the genes already identified are MSX1, PAX9, and AXIN. There is evidence to suggest that PAX9 and MSX1 interact during tooth development [7]. Interaction of such genes along with epigenetic and environmental factors may together be important in determining the expression of this trait. Hence the etiology can be described as being multifactorial and may account for the different teeth missing in the affected individuals of the same family with the same mutation [9].

In the present cases, more teeth were missing in the mandible than the maxilla with more or less bilateral symmetry. There was no consistent pattern of agenesis in various tooth groups except for the third molars. Studies have shown that the most common pattern in the lower arch involved agenesis of all mandibular premolars. Other common patterns in the lower arch were agenesis of the incisors, canine, both premolars, and the second molar. In the maxilla, the most common patterns involved were agenesis of the maxillary lateral incisor and both premolars, whereas in the mandible it was agenesis of all mandibular premolars [2].

A recent study showed that tooth agenesis may be a symmetrical phenomenon [10]. The relatively high-degree of left versus the right-side symmetry of the patterns may indicate a possible common genetic cause. The relatively low-symmetry of upper versus lower arch tooth agenesis may suggest that different mechanisms are responsible for tooth agenesis in the upper and lower arches.

With regard to the association of the location of hypodontia among the siblings, the similarity amongst offspring may indicate that hypodontia affecting different tooth types is caused by different genetic factors. However, the differences of offspring expressing hypodontia in different locations to each other suggest that the combinations of genes and the epigenetic and environmental factors are important in determining expression of hypodontia.

A reduction in tooth dimensions which is as appreciable in siblings in the present case has been documented by previous studies in cases of hypodontia as well as oligodontia. Continuous traits such as height and tooth size typically have a multifactorial mode of inheritance. Brook and Bailit suggested that hypodontia is an example of a “quasicontinuous” trait with a threshold mechanism. The accepted explanation of discontinuous multifactorial variation rests on the assumption that there is an underlying scale of continuous variation, this being, in the case of hypodontia, tooth size. The distribution curve of relatives appears to be to the left of that of the general population. The position of an individual on the scale depends upon a combination of numerous genetic and environmental factors. Proportionally more family members of index patients exceed the threshold for tooth agenesis. The model also suggests that first-degree relatives of index patients will have reduced mean tooth dimensions compared to the general population, even if they do not have hypodontia [11–13].

Dens invaginatus of different grades is appreciated in the central and/or lateral incisors of the siblings which were bilaterally present. Dens invaginatus is most frequently seen in maxillary lateral incisors, but other teeth involvement have also been reported [14]. Some evidence suggests that the problem may be symmetrical [15].

The absence of certain molecules can result in abnormally shaped teeth as well as defects in the developing tooth germ. For this reason the proposal that genetic factors may be the cause of that dens invaginatus has some credibility. The support comes from the study conducted by Grahnen et al. on 3020 Swedish children that reported 2.7% of patients with dens invaginatus, and 43% of their parents and 32% of siblings also had evidence of the condition. Additional support for a genetic influence is drawn from the fact that the invaginations appear to have a limited variation and can occur in a number of teeth in the same individual or in siblings [15]. This partly explains the consistent findings of multiple dens invaginatus in combination with oligodontia in the present cases.

Causative genes, MSX1 and PAX9, can be proposed by the observation that mutations of these genes cause familial and sporadic forms of selective tooth agenesis. PAX9 defect was shown to be responsible for “molar oligodontia”. In contrast, the developmental absence of maxillary and mandibular second bicuspids and maxillary first bicuspids, whilst most mandibular first bicuspids are retained, appears to be the pattern of tooth agenesis that best indicates the presence of an MSX1 mutation [16].

 The changes are that the increasing number of missing permanent teeth cause is limited to the dental and soft tissue parameters. As the number of missing teeth increased, there was a trend for a decrease in the mandibular plane angle, an increase in the facial axis, and a reduction in the lower anterior facial height. This has not been reported in studies with lower prevalence of hypodontia. The typical dentofacial structure seen in persons with severe hypodontia is due to dental and functional compensation rather than to an altered growth pattern [17].

The unesthetic appearance, missing teeth, and overclosure in patients with oligodontia may cause depression and psychosocial problems. Therefore, the main treatment goal is to improve appearance, mastication, and speech. Treatment in such cases is typically multidisciplinary. A wide and expanding range of prosthetic, orthodontic, restorative and surgical therapies are currently employed. The prosthetic treatment of oligodontia varies and includes removable partial dentures, fixed partial dentures, attachment dentures, and overdentures. The choice is dependent on the condition of the remaining teeth [5]. From the orthodontic aspect, the consequences of missing teeth are numerous and depend on the number and type of teeth that are missing. Surgical therapy for such patients can include various aspects, such as autotransplantation, insertion of osteointegrated implants, or orthognathic surgery [18]. Commonly a combined orthodontic-restorative-surgical approach is necessary with orthodontic treatment needed to relocate space in preparation for later conventional fixed prostheses or implants [19].

Although dens invaginatus is common, it may be easily overlooked because of absence of any significant clinical signs of the anomaly. This is unfortunate as the presence of an invagination is considered to increase the risk of caries, pulpal pathosis, and periodontal inflammation. Treatment ranges from conservative restorative procedures (if diagnosed early) to nonsurgical root canal therapy, surgery, or extraction. Root canal treatment of such teeth is often complicated by the unusual forms and location of invaginated and pulpal spaces that complicate thorough debridement [15].

## 4. Conclusion

 The consequences of missing teeth are numerous and depend on the number and type of teeth that are missing. Most frequently speech and masticatory functional disorders occur and, the aesthetic problems are caused by disturbed growth and development of the orofacial area, which can manifest outside the mouth. In the case of patients with oligodontia, prompt and accurate diagnosis is necessary as well as the careful planning of treatment, with a preconception of the final solution. This can only be achieved by multidisciplinary cooperation, which usually includes the following specialists: orthodontist, endodontist, oral surgeon, and prosthetist.

To conclude, it is suggested that detailed studies of oligodontia cases are required to investigate the prevalence and location of missing teeth, the size and morphology of remaining teeth in the dentition, and also the pattern of tooth size, shape, and number in relatives of affected individuals. Such studies may aid in better understanding of underlying factors that are involved in the pathogenesis. The presentation of the dentition in oligodontia being very heterogenous, formulation of universal treatment strategy could be a challenge as homogenous, comparable subgroups of patients are not available.
